# A purinergic call to arms from dying brown adipocytes

**DOI:** 10.15698/cst2022.07.269

**Published:** 2022-07-25

**Authors:** Mohammed K. Hankir

**Affiliations:** 1 Department of Experimental Surgery, University Hospital Wuerzburg, Wuerzburg, 97080, Germany.

**Keywords:** Apoptosis, Obesity, Inosine, Adipose tissue thermogenesis, ENT1

## Abstract

Brown adipocytes react to temperature and nutritional challenges by ramping up their metabolism and generating heat. This adaptation to changes in the environment is crucial for defending organismal homeostasis, but is impaired in obesity and during aging. Writing in *Nature*, Niemann *et al.* show that brown adipocytes become apoptotic under thermoneutral conditions and release ATP, which in turn is converted extracellularly into inosine. They further present evidence that pharmacological and genetic manipulations that enhance signalling of this purine metabolite stimulates thermogenesis in brown adipocytes and promotes metabolic health.

The global incidence of obesity along with its comorbidies such as type 2 diabetes and fatty liver disease continues to rise [1]. This contributes to millions of preventable deaths annually and has major socioeconomic ramifications [2]. In attempts to stem the tide of the obesity pandemic, significant effort has been invested in developing new and improved weight loss drugs [3]. Fortunately, the future doesn't look so bleak since decades of preclinical and clinical research is finally beginning to bear fruit. For example, only recently has the stable gut hormone analogue semaglutide been approved as an obesity treatment [3]. Once-weekly injections of this drug to individuals with obesity can induce on average approximately 20% weight loss after one year [4], enough to improve metabolic health in a major way [5]. Notably, as with most other current obesity drugs [3], semaglutide appears to achieve its effects on body weight by reducing calorie intake through targetting feeding centres in the brain [6].

Since weight gain results from a combination of increased energy intake and reduced energy expenditure, a way of targeting the latter process could further optimize obesity treatments. Thermogenic adipose tissue has attracted much interest in this regard as its activation increases energy expenditure and promotes weight loss in preclinical studies [7]. While appreciable amounts of thermogenic adipose tissue exist in adult humans as well [8], potent activators such as the selective beta-3 adrenergic receptor agonist mirabegron have so far failed to live up to the hype [9, 10]. What's more, thermogenic adipose tissue declines with age and increasing BMI [11, 12], thus approaches that reverse this phenomenon might be needed if effective weight loss by increasing adipose tissue thermogenesis is to be made feasible. In principle, this can be achieved by reversing the cellular scenecence, or cessation of cell division, that occurs in thermogenic adipocyte precursors during aging [13].

Writing in *Nature*, Niemann *et al.* [14] performed a comprehensive set of experiments starting with the relatively neglected subject of apoptosis, or programmed cell death, in thermogenic brown adipocytes. Remarkably, it took just 3 days of inactivating brown adipose tissue thermogenesis by housing mice at their thermoneutral temperature of 30°C to detect apoptotic cells. Fluorescence activated cell sorting then revealed that most of the apoptotic cells in brown adipose tissue under these conditions were indeed brown adipocytes rather than endothelial or immune cells. While apoptosis has been shown to remain elevated in brown adipocytes after prolonged periods of thermoneutrality (4 weeks) [15], it would have been interesting to see if this is also the case in diet-induced obese mice as well as in aged mice.

Niemann *et al.* [14] next asked the intriguing question of what metabolites apoptotic brown adipocytes could release. Through a combination of targetted and untargetted metabolomic approaches, 3 purine metabolites (AMP, inosine and hypoxanthine) were identified to be at 5-10-fold higher concentrations in the media of brown adipoyctes made apoptotic by radiation (UV light) or chemical (nutlin-3) means. This effect was relatively specific to brown adipocytes, as apoptotic endothelial cells and fibroblasts did not show the same pattern or magnitude of purine metabolites in their culture media. Importantly, ectonucleoside triphosphate diphosphoydrolase1 (ENTPD1) and ecto-5'-nucloetidase (NT5E), the enzymes that sequentially dephosphorylate ATP to adenosine, were enriched in brown adipocytes compared to white adipocytes, as was adenosine deaminase (ADA), which next generates inosine in the catalytic cascade, although it would have been interesting to see if the expression/activity of these enzymes increases in apoptotic brown adipocytes. Nevertheless, the data strongly support the idea that the increased release of ATP from dying brown adipocytes serves as the starting point for increased extracellular inosine production.

Niemann *et al.* [14] then proceeded by asking if the metabolites released from apoptotic brown adipocytes communicate with neighbouring healthy brown adipocytes in a paracrine fashion. To do so, an elegant experiment was designed in which the media of apoptotic brown adipocytes was applied onto healthy brown adipocytes. Strikingly, instead of following the same fate as their neighbours, healthy brown adipocytes reacted by increasing thermogenic gene expression suggesting that a ”replace-me” signal is released by apoptotic brown adipocytes. To determine if inosine could be part of this call to arms from dying brown adipocytes, they measured intracellular levels of cAMP, the major second messenger in brown adipocytes that initiates thermogenesis, in response to inosine treatment and assessed downstream signalling of PKA through phosphoproteomic analysis. Not only were intracellular cAMP levels increased by approximately two-fold in inosine-treated brown adipocytes, but of the greater than 8,000 phosphosites regulated by inosine treatment, PKA target sites were among the most represented. Importantly, major downstream targets of PKA in the thermogenic program of brown adipocytes were phosphorylated including mitogen activated protein kinase (MAPK) and cyclic response element binding protein (CREB) which was confirmed by immunoblot analysis. Accordingly, inosine increased mRNA expression of the key thermogenic protein uncoupling protein 1 (UCP1) by approximately two-fold in brown adipocytes and by no less than two orders of magnitute in white adipocytes. The latter finding suggests that inosine promotes the transdifferentiation of energy-storing white adipocytes to so-called thermogenic beige adipocytes. Moreover, inosine stimulated oxygen consumption in isolated brown adipose tissue preperations and in mice. Adenosine and inosine are ligands of 4 GPCRs: the Gi-coupled A1 and A3 receptors and the Gs-coupled A2_A_ and A2_B_ receptors. Consistent with the increase in PKA signalling caused by inosine in brown adipocytes, its thermogenic effects were lost in A2_A_ and A2_B_ receptor knockout mice.

Having established a clear acute thermogenic effect of inosine, Niemann *et al.* [14] then asked if chronic inosine treatment promotes a negative energy balance. Two approaches were taken in which inosine was administered for 4 weeks to mice either before or after the onset of high-fat diet-induced obesity. While inosine modestly but significantly protected lean mice from weight gain on a high-fat diet, it caused marked weight loss (approximately 10%) in mice with established obesity and reduced their blood glucose levels attesting to its therapeutic potential. In both cases, energy expenditure increased and there was increased expression of UCP1 protein in white and/or brown adipose tissue with no associated changes in food intake. These results suggest that inosine mainly targets peripheral tissues to achieve its effects on body weight.

At this junction of the manuscript, Niemann *et al.* [14] were ideally placed to ask how extracellular levels of inosine are regulated by adipocytes. Instead of focusing on Pannexin1 channels that mediate ATP release by apoptotic cells [16], focus was placed on the equilibrative nucleoside transporters 1 and 2 (ENT 1 and 2, respectively). These transporters would hypothetically decrease extracellular inosine levels by transporting it back into cells much like the synaptic reuptake of neurotransmitters in the brain. It was found that ENT1/*Slc29a1* was the most highly expressed of the two transporters in all adipose tissue depots analysed (especially brown adipose tissue) and was indeed required for the uptake of radioactively labelled inosine by brown adipocytes. In line with the hypothesized role of ENT1 in brown adipocytes, extracellular inosine levels were increased under ENT1 deficiency. Remarkably, oxygen consumption was increased in ENT1-deficient brown adipocytes and brown adipose tissue explants. Furthermore, ENT1-deficient brown and white adipocytes had increased thermogenic gene expression and nutrient consumption. These results clearly demonstrate that ENT1 serves to clear extracellular inosine in brown adipose tissue to put a brake on thermogenesis.

Since chronic inosine treatment was shown to promote a negative energy balance, it would be reasonable to expect that chronic loss of ENT1 function would have a similar effect. To address this question, Niemann *et al.* [14] generated whole-body and adipose tissue-specific ENT1 knockout mice. Both mutant mouse lines showed similar metabolic phenotypes such as increased energy expenditure, protection from weight gain on a high-fat diet, improved glucose tolerance and increased thermogenic gene expression in brown and white adipose tissue. Interestingly, *Slc29a1* mRNA was found to be expressed in platelet-derived growth factor receptor A (PDGFRα) positive stromal vascular cells in white adipose tissue, raising the possibility that inosine promotes the differentiation of thermogenic precursors as well as transdifferentiation of mature white adipocytes mentioned earlier. Despite the largely overlapping phenotypes of global and adipose tissue-specific ENT1 knockout mice, it should be noted that the former were more protected from obesity suggesting that ENT1 in other peripheral tissues, such as skeletal muscle, promotes a positive energy balance.

When delivered systemically, inosine could have negative off-target effects, thus an approach of enhancing its signalling more locally could be safer [17]. Niemann *et al.* [14] therefore tested the approved anti-platelet drug and ENT1 blocker dipyridamole in wild-type mice. Interestingly, for dipyridamole to exert functional effects such as lipolysis, inosine first needed to be released by adipocytes. This could be achieved with noradrenaline and cold exposure as determined by in vivo microdialysis. Moreover, the combination of dipyridamole with a selective beta-3 adrenergic receptor agonist or cold exposure increased oxygen consumption and thermogenic gene expression in white adipose tissue, although it failed to significantly reduce adiposity. Nevertheless, it would be interesting to determine the impact of dipyridamole treatment in the context of diet-induced obesity in future studies.

Finally, to translate the preclinical findings to the clinical context, Niemann *et al.* [14] performed experiments on cultured human brown and white adipocytes. Similar to mouse brown adipocytes, apoptosis increased extracellular inosine levels by approximately 3-fold as did noradrenaline treatment in human brown adipocytes. Inosine also increased thermogenic gene expression in these cells. Knockdown of ENT1 by Crispr-Cas9 in both brown and white human adipocytes increased oxygen consumption and thermogenic gene expression. Consistent with the knockdown experiments in cells, strong negative correlations were found between *SLC29A1* and thermogenic gene expression in subcutaneous and visceral white adipose tissue in a large human cohort of approxmiately 1,500 individuals. Furthermore, analysis of the Genome Aggregation Database revealed that the single nucleotide polymorphism c.647T>C in the *SLC29A1* gene leading to a Ile216Thr substitution in the ENT1 protein occured with high frequency. Functional studies further revealed that this variant reduced inosine uptake in cells by approximately 30%. Remarkably, in a self-contained German population of 895 individuals, the Il2216Thr variant signficantly associated with a lower BMI and homo/heterozygous carriers were more likley to be underweight and healthy, although it remains to be determined whether this is through effects on adipose tissue thermogenic capacity [17].

Since its rediscovery in 2009 [11, 18, 19], thermogenic adipose tissue research has blossomed with new transcription factors, thermogenic pathways and signalling molecules being discovered almost on a daily basis. The study of Niemann *et al.* [14] has expanded our understanding of brown adipocytes by revealing that as they die, they release an abundance of signalling molecules including purine metabolites. However, while there is overwhelming evidence that increasing inosine levels, either pharmacologically or genetically by deleting ENT1, promotes adipose tissue thermogensis and metabolic health, it remains unclear to what extent it contributes to adipose tissue function under apoptotic conditions. There are several ways that this can be addressed. For example, while Niemann *et al.* [14] found that applying the culture media of apoptotic brown adipocytes to healthy brown adipocytes increases thermogenic gene expression, it was not shown whether this was prevented by blocking A2_A_ and/or A2_B_ receptors which would have provided evidence for the role of inosine in mediating this effect. Also, it would be interesting what effects blocking inosine signalling has on adipose tissue under thermoneutral conditions or in aged mice when apoptosis is expected to be high. If dying brown adipocytes do indeed make a purinergic call to arms to neighbouring healthy brown adipocytes, blocking inosine signalling under such conditions should diminish thermogenesis even further. Notably, adipose tissue-specific A2_B_ receptor knockout mice show normal energy expenditure when acutely housed at thermoneutrality [20], although this might be different in the chronic setting (greater than 3 days). These outstanding questions aside, the groundbreaking study of Niemann *et al.* [14] provides new insight into brown adipose function in health and disease and pave the way forward for developing innovative new treatments for obesity by promoting adipose tissue thermogenesis.
